# Gastrointestinal Parasites in Pigs in Southern Mozambique: Prevalence, Diversity and Their Importance in Public and Animal Health

**DOI:** 10.3390/pathogens15070750

**Published:** 2026-07-17

**Authors:** Célio Alfredo, Lurdes Delgado, Lúcel Fernandes, Omar Manito Mavilingue, Ilídio Filipe Manuel, António Castro, Helder Cortes

**Affiliations:** 1 Department of Veterinary Medicine, Faculty of Veterinary Medicine and Animal Science, Save University, National Road No. 1, Parcel 76, Chongoene 1200, Gaza, Mozambique; 2 MED—Mediterranean Institute for Agriculture, Environment and Development & CHANGE—Global Change and Sustainability Institute, IIFA, Universidade de Évora, 7006-554 Évora, Portugal; 3 Centro de Estudos de Ciência Animal, Fundação Fernando Pessoa, Avenida Fernando Pessoa, n.° 150, 4420-096 Gondomar, Portugalantonio.castro@insa.min-saude.pt (A.C.); 4 Instituto Nacional de Saúde Doutor Ricardo Jorge, INSA-Porto, 1649-016 Lisbon, Portugal; 5 MED—Mediterranean Institute for Agriculture, Environment and Development & CHANGE—Global Change and Sustainability Institute, Laboratório de Parasitologia Victor Caeiro, Departamento de Medicina Veterinária, Escola de Ciência e Tecnologia, Universidade de Évora, 7006-554 Évora, Portugal

**Keywords:** parasites, One Health, swine, epidemiology, Mozambique

## Abstract

**Background:** Pigs serve as reservoir hosts for various gastrointestinal parasites, posing significant implications for livestock productivity and public health. Data on gastrointestinal parasites in pigs in Mozambique are scarce, while previous studies have focused mainly on porcine cysticercosis. This study aimed to assess the prevalence and diversity of gastrointestinal parasites in pigs of southern Mozambique. **Methods:** A cross-sectional epidemiological study was conducted with 339 fecal samples, corresponding to the same number of pigs, one sample per animal. Ritchie concentration and Ziehl-Neelsen staining methods were used to detect parasites. **Results:** An overall prevalence of 88.5% (300/339; 95% CI: 85.1–91.9) was obtained. Eight distinct parasites were identified, with Coccidia exhibiting the highest prevalence at 64.3% (218/339), Strongyle-type eggs at 32.5% (109/339), *Cryptosporidium* spp. at 26.6% (90/339), *Entamoeba* spp. at 25.1% (85/339), *Balantioides coli* at 22.7% (77/339), *Ascaris suum* at 17.4% (59/339), *Giardia* spp. at 14.2% (48/339), and *Trichuris* spp. at 5.6% (19/339). None of the variables analyzed showed a significant association with the infection of pigs (*p* > 0.05). **Conclusions:** This is the first large-scale, multi-parasitic epidemiological investigation of gastrointestinal parasites in pigs across southern Mozambique. The detection of *Giardia* spp., *Cryptosporidium* spp., *Balantioides coli*, and *Entamoeba* spp. underscores the need for molecular methods to clarify the zoonotic potential of these parasites in the region.

## 1. Introduction

Pigs serve as reservoir hosts for a wide range of gastrointestinal parasites, with significant implications for both livestock productivity and public health [1,2,3]. While infections in pigs are frequently asymptomatic, under certain conditions, they can result in substantial economic losses, including impaired feed conversion efficiency, reduced reproductive performance, decreased litter size, lower weaning weights, and increased carcass condemnation rates at slaughter [3,4,5,6,7,8].

Furthermore, it is widely documented that parasitized pigs exhibit increased susceptibility to secondary infectious and non-infectious diseases, further compromising animal health, welfare, and production efficiency [9,10]. Among the parasites that cause infections in pigs, those that have been detected most frequently include the protozoa *Balantioides coli*, Coccidia, *Entamoeba* spp., and *Cryptosporidium* spp., as well as the nematodes *Ascaris suum* and *Trichuris suis* [11]. Several of these parasites, including *Balantioides coli*, *Entamoeba* spp., *Cryptosporidium* spp., and *Giardia duodenalis*, possess zoonotic potential and represent significant public health threats. The close spatial proximity between humans and pigs in various farming communities facilitates zoonotic transmission of these enteric pathogens, which are frequently implicated in human diarrheal diseases [4,12,13,14].

The complications resulting from infection by these pathogens in animals, as well as the zoonotic risk, are exacerbated due to the course of these infections, which are asymptomatic in the pig population, and, in most cases, breeders are unable to identify the first clinical signs related to infection and, equally, in a few cases consider the involvement of parasitic agents [6,8,9,15].

In Mozambique, knowledge regarding gastrointestinal parasites infecting pigs remains limited, with no comprehensive epidemiological surveillance data available on parasite diversity, prevalence, or associated risk factors in pig populations. The sparse scientific literature on porcine parasitism in Mozambique has focused predominantly on cysticercosis caused by *Taenia solium*, with studies concentrated in Tete Province, central Mozambique [1,16], and limited investigations in Angonia and Boane districts, in the central and southern parts of the country, respectively [17]. Moreover, the potential role of pigs as reservoirs for human infections remains unexplored in Mozambique. Therefore, this study aimed to assess the prevalence, diversity, and geographic distribution of gastrointestinal parasites in pigs raised across southern Mozambique, and to identify risk factors associated with parasitic infections. By providing comprehensive, large-scale epidemiological data, this research seeks to enhance understanding of circulating parasites in pig populations and inform the development of evidence-based prevention and control strategies based on the One Health approach.

## 2. Materials and Methods

### 2.1. Study Area

**Figure 1:**
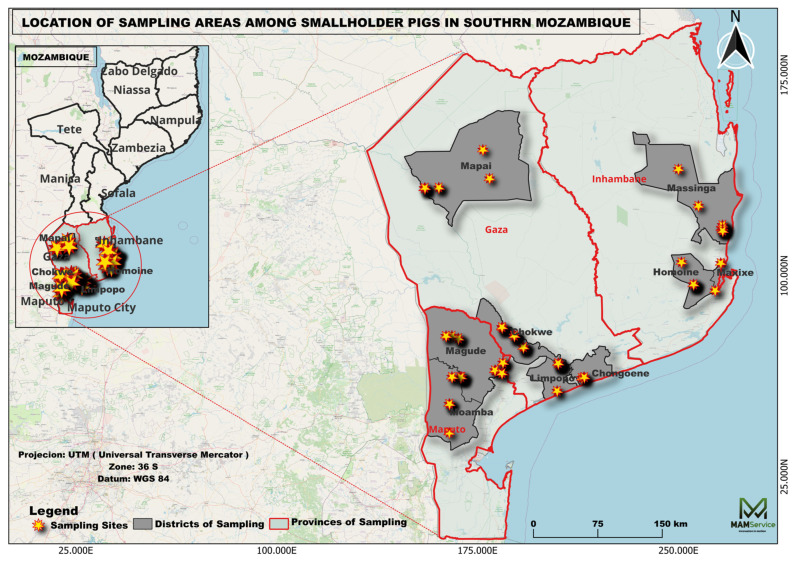
Location of sampling areas among smallholder’s pigs in Southern Mozambique.

This study was conducted in southern Mozambique, encompassing the provinces of Maputo, Gaza, and Inhambane. Sampling was performed in the districts of Magude and Moamba (Maputo Province); Limpopo, Chókwè, Mapai and Chongoene (Gaza Province); and Maxixe, Massinga, and Homoíne (Inhambane Province) (Figure 1). The southern region is characterized by a dry tropical climate, with mean annual temperatures ranging from 22 °C to 27 °C and mean annual precipitation between 400 and 800 mm, concentrated primarily between November and March [18]. The predominant vegetation comprises wooded savannas, dry forests, and natural grasslands interspersed with agricultural and forestry zones.

Within the country, pig farming varies by region and according to producers’ financial capacity. The largest national herd is found in the central region, particularly in Tete Province, with approximately 441,843 animals. In the south, the largest production is concentrated in the province of Gaza, with approximately 206,609 animals, while in the north, the province of Nampula has a herd estimated at 94,416 pigs [19].

In general, pig farming in Mozambique is organized into three production systems: extensive (free-range), semi-intensive, and intensive. The main breeds raised are Landim (native) and imported breeds such as Large White, Landrace, and Duroc. In the southern region and across the country in general, pig production by small-scale farmers is significantly limited by diseases such as African swine fever (ASF), *Taenia solium* cysticercosis (PC), and other parasitic diseases, as well as low farming skills, general poverty, and the lack of a market network for pigs [1,20,21].

In addition, farmers’ limited knowledge of disease management and prevention [22], insufficient and poor-quality feed, the scarcity of veterinary and extension services [23], as well as the lack of adequate housing and confinement [24], pose additional challenges for pig farming in Africa, including Mozambique.

### 2.2. Study Design and Sampling

A cross-sectional epidemiological study was conducted across southern Mozambique to determine the prevalence and diversity of gastrointestinal parasites circulating in pig populations. Additionally, farm-level epidemiological data were collected to identify risk factors associated with parasitic infections.

District selection employed stratified random sampling, with the three southern provinces (Gaza, Inhambane, and Maputo) serving as primary strata. Four districts were randomly selected from each province for inclusion in the study. However, due to post-election civil unrest in Mozambique between October 2024 and January 2025, sampling campaigns in Boane, Matutuíne, and Vilankulos districts could not be completed, resulting in their exclusion from the final study design. Within each selected district, pig farm lists were obtained from District Economic Activities Services (SDAE). Farms were assigned unique identification codes and entered into a database, from which production units were randomly selected using computer-generated random numbers. Following farm selection, producers were contacted to obtain informed consent, and fecal samples were subsequently collected from participating farms. In each production unit, one-third of the pigs on the farm were included to ensure sample representativeness. This proportion was defined based on the characteristics of pig production systems in the southern region of the country, which are predominantly composed of small-scale farmers maintaining herds of 5 to 20 animals. Therefore, the inclusion of one-third of the animals from each farm was considered sufficient to generate generalizable information for each selected unit. Subsequently, animals on each farm were selected in a homogeneous manner, ensuring representativeness across sex and age groups.

### 2.3. Collection and Analysis of Fecal Samples

The study was conducted between October 2024 and May 2025, during which 339 fecal samples were collected from pigs raised in smallholder family farms. Samples were obtained directly from the rectal ampulla using latex gloves, with approximately 10–30 g of feces collected per animal. Following collection, the samples were identified and packed in insulated boxes with ice packs, and transported to the Parasitology Department of Chongoene Health Center, Mapai District Laboratory, and Chicuque Rural Hospital Laboratory (Maxixe), for initial detection of the parasites. Subsequently, prepared slides were transported to the Biology Laboratory at Save University for staining procedures.

Laboratory analysis employed two parasitological techniques: (1) the Ritchie concentration technique (formalin-ethyl acetate sedimentation, with modifications) for detection of parasite cysts, oocysts, and helminth eggs; and (2) modified Ziehl-Neelsen (acid-fast) staining for identification of *Cryptosporidium* spp. oocysts.

### 2.4. Centrifugal Sedimentation Technique (Ritchie)

The Ritchie concentration technique was performed following the protocol described by [25] with minor modifications, specifically with regard to the use of 10% formalin, which was replaced with distilled water. Approximately 3 g of fecal material was homogenized in 15 mL of distilled water and filtered through sterile gauze. The filtrate was transferred to a graduated conical centrifuge tube and the volume was adjusted to 14 mL with distilled water. The tube was centrifuged (Manufacturer: Jinan Biobase Medical Co. LTD, Model: BKC-TL4MII, Serial number: LXJTL422020086K, Country: China) at 1500 rpm for 5 min, and the process was repeated with fresh distilled water until the supernatant became clear. Following clarification, 10 mL of distilled water and 4 mL of diethyl ether were added to the sediment, and the tube was vigorously shaken for 10 s to ensure thorough mixing. The tube was then centrifuged under identical conditions, producing four distinct layers: ether (top), debris, aqueous phase, and sediment (bottom). The supernatant layers were carefully decanted while preserving the final sediment. An aliquot of resuspended sediment was transferred to a glass microscope slide and covered with a coverslip for microscopic examination. Parasitic elements (cysts, oocysts, and helminth eggs) were identified based on morphological characteristics using bright-field microscopy at 10× and 40× magnifications [26].

### 2.5. Ziehl-Neelsen Staining

For the detection of *Cryptosporidium* spp., stool smears were obtained from the sediment using the Ritchie technique, spread on microscopy slides, and dried at room temperature (37 °C) [27]. The slides were then fixed with methanol for 30 s and stained with carbol fuchsin for 1 min. After staining, the slides were washed with distilled water, soaked in an acid-alcohol solution for 2 min, then washed again with distilled water. Malachite green was applied for 2 min, and the slides were washed and dried at room temperature.

To visualize *Cryptosporidium* spp. oocysts, the samples were observed under light microscopy (model BA310, Motic, Xiamen, China) using an immersion objective (100x). The slides were read by two independent individuals and confirmed by a third microscopist. Due to difficulties in obtaining reference slides in the region, the identification of *Cryptosporidium* spp. oocysts was based on veterinary parasitology slides provided by the Department of Veterinary Medicine, University of Évora, Portugal, where the parasite appears pink to red in color, spherical to ovoid in shape, with bodies and a bluish background [28].

### 2.6. Statistical Analysis

All data on the animals and the laboratory results were recorded on forms designed for this purpose and then entered into Microsoft Excel, Windows 10. At a later stage, all the information was exported to the Statistical Package for the Social Sciences (SPSS Inc., Chicago, IL, USA), version 23.0, to estimate the prevalence of gastrointestinal parasites, as well as to cross-reference the data with sociodemographic variables of the production units, such as: age, sex, and frequency of deworming, veterinary care, and regular removal of feces. Fisher’s exact test and the odds ratio (OR) were used to determine the main factors associated with infection of pigs by the enteroparasites considered in this study, adopting a significance level of 0.05.

## 3. Results

### 3.1. Farm Characteristics

A total of 142 smallholder pig farms were sampled across nine districts in Gaza, Maputo, and Inhambane provinces in southern Mozambique, yielding 339 fresh fecal samples. The distribution of samples was as follows: 174 (51.3%) from Gaza Province, 95 (28.0%) collected in Maputo Province, and 70 (20.6%) were obtained in Inhambane Province. The sampled population consisted predominantly of female pigs (55.7%, 189/339) compared to males (44.3%, 150/339), with adults representing the largest age category (48.9%, 166/339). Regarding pig-raising systems, most farms, 94 (66.2%), operated under extensive production. An additional 40 farms (28.2%) used a semi-intensive system, while the remaining 8 farms (5.6%) practiced intensive production.

Farm infrastructure was generally rudimentary, characterized by wooden structures without concrete flooring, with a mean herd size of six pigs per farm. The majority of farmers (80.3%, 114/142) reported no access to veterinary services, and most production facilities did not deworm their animals (83.8%, 119/142). Regular fecal removal from pig housing was infrequently practiced, with 53.5% (76/142) of farms lacking routine stool removal. Additionally, 41.5% (59/142) of farmers applied pig feces directly to vegetable fields as fertilizer (Table 1).

### 3.2. Prevalence of Gastrointestinal Parasites

**Figure 2:**
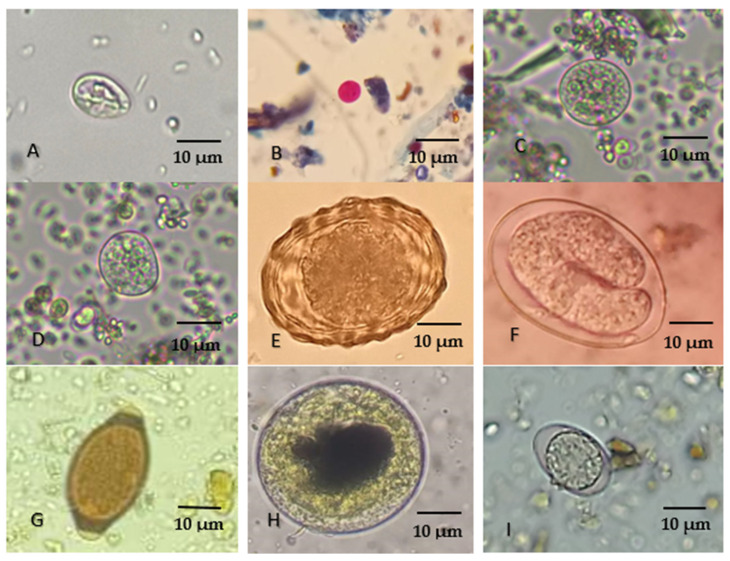
Detected parasites in pigs of southern Mozambique. (**A**)—*Giardia* spp., (**B**)—*Cryptosporidium* spp., (**C**,**D**)—*Entamoeba* spp., (**E**)—*Ascaris suum*, (**F**)—Strogyle-type, (**G**)—*Trichuris* spp., (**H**)—*Balantioides coli*, (**I**)—Coccidia.

Of the 339 fecal samples analyzed, 300 tested positive for at least one gastrointestinal parasite, with an overall prevalence of 88.5% (300/339; 95% CI: 85.1–91.9%). Eight distinct parasites were identified, with Coccidia exhibiting the highest prevalence at 64.3% (218/339), followed by Strongyle-type eggs at 32.5% (109/339), *Cryptosporidium* spp. at 26.6% (90/339), *Entamoeba* spp. at 25.1% (85/339), *Balantioides coli* at 22.7% (77/339), *Ascaris suum* at 17.4% (59/339), *Giardia* spp. at 14.2% (48/339), and *Trichuris* spp. at 5.6% (19/339) (Figure 2 and Table 2).

### 3.3. Distribution of Infections

Regarding the distribution of infections by collection site, it was found that the infection rate was highest in Gaza Province, at 90.2% (157/174), followed by Maputo Province at 89.5% (85/95), and finally Inhambane Province with a prevalence of 82.8% (58/70). It was also noted that of the nine districts where samples were collected, the prevalence of infection was highest in the districts of Chongoene (Gaza Province) and Magude (Maputo Province), at 48.9% (85/174) and 56.8% (54/95), respectively.

Female pigs demonstrated higher infection prevalence (49.3%, 167/339) compared to males (39.2%, 133/339), although this difference was not statistically significant (*p* = 0.92). By age category, adult animals exhibited the highest infection prevalence at 42.8% (145/339). Breed-specific analysis revealed that Landim pigs had the highest infection rate at 78.3% (235/300), followed by Large White at 11.0% (33/300) and Crossbred animals at 10.6% (32/300).

Management practices analysis indicated higher infection prevalence among animals not routinely dewormed (84.3%, 253/300) compared to those receiving regular anthelmintic treatment (15.7%, 47/300), although this difference was not statistically significant (*p* = 0.64). Similarly, infection prevalence was higher among animals without access to veterinary care (82.3%, 247/300) compared to those receiving veterinary services (17.7%, 53/300), with no statistically significant difference observed (*p* = 0.72). Farms that did not practice regular fecal removal exhibited higher positivity rates (56.7%, 170/300) compared to those with routine feces removal (43.3%, 130/300), although this association was also not statistically significant (*p* = 0.37).

### 3.4. Risk Factors

Risk factor analysis evaluated eight variables potentially associated with gastrointestinal parasitic infection: animal sex, age category, anthelmintic treatment frequency, access to veterinary services, feces removal practices, pen disinfection frequency, geographic origin, and breed. Although higher infection prevalence was observed among female pigs, non-dewormed animals, Landim breed pigs, and farms lacking regular fecal removal and veterinary services, none of these variables demonstrated statistically significant associations with infection status (*p* > 0.05), Table 3.

## 4. Discussion

This study documented an overall prevalence of gastrointestinal parasites of 88.5% in pigs across southern Mozambique, representing one of the highest rates reported in swine populations globally. This elevated prevalence likely reflects suboptimal management conditions characteristic of smallholder production systems in the region, including limited veterinary services, absence of routine anthelmintic treatment, inadequate feces removal, and feeding of spoiled food waste consisting primarily of human food scraps. The infection rate observed in this study is consistent with findings from comparable epidemiological investigations in other developing regions, which have reported prevalence rates of 90.4% in Argentina, 91.1% in Ghana, 85.2% in Thailand, and 84.6% in Rwanda [3,7,8,29], suggesting a common epidemiological pattern of gastrointestinal parasitism in resource-limited pig production systems.

Among the eight parasites identified in this study, *Cryptosporidium* spp. *Entamoeba* spp., *Balantioides coli*, and *Giardia* spp. have particular attention because studies conducted in various parts of the world have reported the circulation in pigs of species, genotypes and variants capable of causing infections in humans [30,31,32,33]. For *Cryptosporidium* (26.6%), although *Cryptosporidium suis* and *Cryptosporidium scrofarum* are the predominant species documented in swine [34], recent molecular epidemiological studies have demonstrated circulation of other species, notably *Cryptosporidium parvum* and *Cryptosporidium muris*, in pig populations [32].

In addition, this concern is particularly relevant in southern Mozambique, where free-range pig management systems are widely practiced in some districts. In these systems, pigs are released daily to forage and subsequently confined to rudimentary housing at night, resulting in extensive pig-human contact, particularly with children, as well as interactions with other domestic animals. This production model facilitates fecal-oral transmission of the parasites between animals and humans. The situation is further compounded by the widespread practice of applying untreated pig feces directly to vegetable fields, which was observed on 41.5% of farms surveyed. This practice poses significant food safety risks, as crops such as lettuce consumed raw may become contaminated with parasitic oocysts and other enteric pathogens.

In humans, cryptosporidiosis presents with variable clinical severity, ranging from mild, self-limiting gastroenteritis to severe, persistent diarrhea accompanied by abdominal cramping, nausea, anorexia, and weight loss. The infection poses particular risks for immunocompromised individuals, in whom it may progress to chronic disease with life-threatening hydroelectrolytic imbalances. In swine and other livestock, *Cryptosporidium* infections are typically subclinical; however, clinical disease in neonatal piglets can result in diarrhea, growth retardation, and increased mortality, negatively impacting production efficiency [35].

*Balantioides coli* (22.7%), a neglected parasitic ciliate, is maintained primarily in pig reservoirs, although other livestock species including camels, cattle, donkeys, sheep, and goats have been identified as potential sources of human infection [36]. While infections in pigs are predominantly asymptomatic, *B. coli* is a recognized zoonotic pathogen capable of causing sporadic cases and outbreaks of human balantidiasis. Genotype A has been particularly associated with human clinical cases [37,38] and can progress to severe dysentery and ulcerative colitis, which may prove fatal in immunocompromised or malnourished individuals [35,36]. Epidemiological investigations have identified close proximity to pig populations as a significant risk factor for human balantidiasis [14,39], underscoring the importance of proper sanitation and biosecurity measures in pig farming communities.

*Giardia* spp. (14.2%), a flagellated protozoan, is recognized as a major causative agent of diarrheal disease in humans and animals worldwide. In humans, giardiasis manifests as persistent watery diarrhea, abdominal pain, nausea, and nutrient malabsorption, with increased risk of dehydration and malnutrition among vulnerable populations, particularly children and immunocompromised individuals [40].

*Giardia duodenalis* is currently recognized as a species complex comprising eight genetically distinct assemblages (A–H) that exhibit varying degrees of host specificity. Human infections are predominantly caused by assemblages A and B. In dogs and cats, assemblages C/D and F predominate, respectively, while assemblage E is most commonly associated with ruminant infections [41,42]. Molecular epidemiological studies in swine have documented six assemblages (A–F) capable of infecting pigs. According to systematic review and meta-analysis data, assemblage E is most frequently detected in pigs (41.1%), followed by assemblages B (28.2%), D (16.2%), C (11.6%), and A (9.9%), while assemblage F has been reported in only a single investigation [43].

*Entamoeba* spp. exhibited an overall prevalence of 25.1%. While there is limited evidence supporting the occurrence of *Entamoeba histolytica* in pigs, recent molecular studies have identified circulation of other species, notably *Entamoeba polecki*, which comprises subtypes with documented zoonotic potential, particularly in cases of co-infection with other enteric pathogens [33,44,45,46].

Although the study reported high prevalences and diversity of gastrointestinal parasites in pigs, molecular epidemiological data would certainly be valuable for identifying species, genotypes, and variants. However, in the absence of genotyping, the current results do not allow for a robust assessment of zoonotic risk. Therefore, molecular studies are necessary not only for the assessment of zoonotic risk but also for the design of evidence-based parasite control strategies based on the One Health approach in the region. Similarly, future research should prioritize the epidemiological assessment of these enteroparasites in relation to seasonality, in order to identify patterns and periods of peak transmission of these pathogens, with the aim of better targeting parasite control interventions in the swine population.

Coccidia represented the most frequently detected parasitic group (64.3%), consistent with findings from epidemiological studies conducted globally, which have documented similarly high prevalence rates of coccidian parasites in swine populations [47,48,49]. In the present investigation, differentiation between *Eimeria* spp. and *Cystoisospora suis* could not be achieved, representing a methodological constraint of the study. Although these coccidian exhibit diagnostic morphological distinctions following oocyst sporulation, all oocysts recovered in this study were unsporulated at the time of examination. The absence of sporulation consequently limited the differentiation between the two groups of parasites.

While both genera are associated with enteric disease, *C. suis* is considered the primary etiological agent of clinical coccidiosis in suckling piglets, capable of inducing severe hemorrhagic diarrhea, particularly in animals aged 7–21 days, whereas *Eimeria* spp. infections are generally characterized by lower pathogenicity [50,51]. Therefore, we suggest that future studies should prioritize molecular characterization of coccidian parasites circulating in southern Mozambican pigs to generate species-level data and help to inform targeted control strategies for porcine coccidiosis.

Geographic analysis revealed the highest infection prevalence in Gaza Province (90.2%), with Chongoene (Gaza) and Magude (Maputo) districts exhibiting the highest district-level rates at 48.9% and 56.8%, respectively. The elevated prevalence observed in Gaza Province may be partially attributed to sampling process, as 51.3% (174/339) of all samples were collected from this province, which harbors the largest pig population in southern Mozambique, estimated at 206,609 animals [19], and not necessarily due to the influence of factors related to animal handling, since the pattern is practically the same in the three provinces where the sampling campaigns were carried out.

Gastrointestinal parasite positivity was observed more frequently in samples from females than in those from males, although this difference was not statistically significant. Previous epidemiological studies have reported variable patterns regarding sex-associated infection risk. In Nepal, Adhikari, R.B. et al. [52] documented higher prevalence in males (95.7%) than females (87.0%) without statistical significance, while in South Africa, Nwafor, I.C. et al. [49] reported significantly higher infection rates in females (86.7%) compared to males (68.8%) (*p* < 0.05). These inconsistent findings across geographic regions suggest that sex is not an independent predictor of infection risk and that other epidemiological factors play more substantial roles in determining infection dynamics.

Although a higher prevalence was observed in females, in non-dewormed animals of the Landim breed, and in farms where feces were not regularly removed and there was no veterinary assistance, none of these variables showed a statistically significant association with the infection status. This finding suggests that such factors play an important role in the transmission of parasites; however, the lack of significance may have been influenced by the imbalance between the groups evaluated, since a large proportion of breeders do not deworm their animals, do not receive veterinary care, and do not regularly clean the pigsties. This scenario may have reduced the statistical power of the tests and made it difficult to detect significant differences. However, in the study carried out in Mozambique, by [1], results close to ours were obtained, finding that only one factor was associated with the infection of pigs by gastrointestinal parasites, which was the origin of the animals (village).

## 5. Conclusions

This study represents the first large-scale, multi-parasitic epidemiological investigation of gastrointestinal parasites in pig populations across southern Mozambique, providing critical baseline data for the development of control strategies targeting parasites of both veterinary and public health significance. The documented circulation of *Giardia* spp., *Cryptosporidium* spp., *Balantioides coli*, and *Entamoeba* spp. underscores the need for molecular epidemiological investigations utilizing PCR-based methods to characterize species, genotypes, and assemblages of these pathogens. Such studies are essential to assess the zoonotic risk posed by pig-associated parasites and to elucidate transmission pathways between pigs and humans in the region. Veterinary and public health authorities should prioritize educational interventions aimed at improving farmer awareness of parasitic disease risks and promoting adoption of improved management practices, including routine anthelmintic treatment and regular feces removal from pig housing, including actions that encourage changes in behavior, particularly in aspects related to the use of pig feces in vegetable fields, a common practice in the region.

## Figures and Tables

**Table 1:** Management practices and breeding conditions for pigs in southern Mozambique.

Variables Characteristics		Responses
Deworming	No	119 (83.8%)
	Yes	23 (16.2%)
Veterinary assistance	No	114 (80.3%)
	Yes	28 (19.7%)
Regular Stool removal	No	76 (53.5%)
	Yes	66 (46.5%)
Stool use in agriculture	No	83 (58.5%)
	Yes	59 (41.5%)
Sex	Male	150 (44.3%)
	Female	187 (55.7%)
Age	Adult	166 (48.9%)
	Piglet	62 (18.3%)
	Weaner	111 (32.7%)
Breed	Landim	267 (78.8%)
	Large white	38 (11.2%)
	Crossbred animals	34 (10%)
Sample status	Diarrheal	24 (7%)
	Formed	301 (88.8%)
	Pastelike	14 (4.2%)
Source of samples	Gaza province	174 (51.3%)
	Maputo province	95 (28%)
	Inhambane province	70 (20.7%)
Breeding system	Extensive	94 (66.2%)
	Semi-intensive	40 (28.2%)
	Intensive	8 (5.6%)

**Table 2:** Types of parasites detected in pigs in southern Mozambique and their respective prevalence rates.

Parasites	Positive (*n*)	Prevalence (%)	IC-95 (%)	
Strongyle-type	109	32.5	27.2	37.1
Coccidia	218	64.3	59.2	69.4
*Balantioides coli*	77	22.7	18.5	27.6
*Ascaris suum*	59	17.4	13.4	21.4
*Trichuris* spp.	19	5.6	3.2	8.1
*Giardia* spp.	48	14.2	10.5	17.8
*Cryptosporidium* spp.	90	26.6	21.8	31.2
*Entamoeba* spp.	85	25.1	20.4	29.7

**Table 3:** Risk factors for gastrointestinal parasite infection in pigs in southern Mozambique.

Variables	Risk Factor	OR	IC 95%	*p*-Value
Deworming	Yes	1.264	0.457–4.348	0.64
	No			
Sex	Male	1.031	0.499–2.158	0.93
	Female			
Age	Adult vs. Piglet	0.93	0.37–2.34	0.95
	Adult vs. Weaner	0.89	0.41–1.94	
	Piglet vs. Weaner	0.95	0.43–2.63	
Veterinary Assistance	Yes	1.18	0.46–3.62	0.71
	No			
Origin	Gaza vs. Maputo	1.09	0.48–2.49	0.25
	Gaza vs. Inhambane	1.91	0.87–4.18	
	Inhambane vs. Maputo	0.57	0.23–1.41	
Stool removal	Yes	1.36	0.65–2.96	0.37
	No			
Breed	Landim vs. Large white	1.11	0.40–3.05	0.54
	Landim vs. Crossbred	0.46	0.11–2.03	
	Large white vs. Crossbred	0.41	0.07–2.27	
Breeding system	Extensive vs. Intensive	2.70	0.93–7.82	0.16
	Extensive vs. Semi-intensive	1.50	0.61–3.68	
	Intensive vs. Semi-intensive	0.56	0.24–1.30	

## Data Availability

The data supporting this investigation can be obtained by requesting them from the study authors and obtaining authorization from the Provincial Directorates of Fisheries and Agriculture of Maputo, Gaza, and Inhambane.
